# Genetic Variation of PPARs

**DOI:** 10.1155/2009/189091

**Published:** 2009-12-01

**Authors:** Marie-Claude Vohl, Mostafa Badr, Stefan Wieczorek

**Affiliations:** ^1^Lipid Research Center, CHUL Research Center, Laval University, Quebec City, Canada G1V 4G2; ^2^Division of Pharmacology, School of Pharmacy, University of Missouri-Kansas City, MO 64108, USA; ^3^Human Genetics Department, Ruhr-University Bochum, 44801 Bochum, Germany

Welcome to this special issue of PPAR Research dedicated to the “Genetic Variation of PPARs.” Since PPARs are nuclear transcription factors regulating multiple genes involved in energy production, glucose and lipid metabolism, polymorphisms in these receptors may influence the pathology of numerous diseases including obesity, diabetes, atherosclerosis, inflammation and cancer.

The first section of this special issue of PPAR Research contains a series of four original research articles followed by a review article examining the impact of *PPAR* gene polymorphisms on various metabolic diseases. First is an article by Deeb and Brunzell describing the impact of the Gly482Ser polymorphism in the PPARG coactivator-1 alpha (*PPARGC1A*) on weight gain in a diabetic population. A second article by Dallongeville and coworkers examines the association of *PPARG* gene polymorphisms with coronary heart disease. Third, Wieczorek's research group investigates the consequences of polymorphisms in *RXRB*, *PPARA*, and *PPARG* on Wegener's Granulomatosis. Fourth, a study by Ereqat et al. presents the results of an investigation of the impact of the *PPARG* Pro12Ala polymorphism on the metabolic and clinical characteristics in Palestinian type 2 diabetic patients. Finally this section ends with a review by Weimin He on the influence of the *PPARG* Pro12Ala polymorphism on insulin sensitivity, as well as other diseases including cancer, polycystic ovary syndrome, Alzheimer disease, and aging. 

We are also pleased that this special issue contains two articles that describe the functional effects of the *PPAR* gene polymorphisms. First, Rudkowska and co-researchers describe the differences in transcriptional activation observed in two allelic variants of *PPARA* (L162V) after omega-3 fatty acids treatment. Second, McCleelland et al. discern the regulation of translational efficiency by disparate 5′ UTRs of *PPARG* splice variants. These two articles lead to a more complete understanding of the role and functional repercussions of various *PPAR* gene polymorphisms in the prevention and treatment of diseases. 

In conclusion, while the influence and impact of PPAR polymorphisms on health and disease is still mostly uncertain, recent evidence suggests that these genetic variations play an important role in the initiation/progression of disease as well as in the efficacy of specific treatments in particular individuals/populations. We are fortunate to have received contributions from such well-renowned experts in the field, and hope that you will find that this special issue of PPAR Research produces greater interest in this critical and evolving field of research. 

*Marie-Claude Vohl* *Marie-Claude Vohl* *Mostafa Badr* *Mostafa Badr* *Stefan Wieczorek* *Stefan Wieczorek*

